# Clinical and Laboratory Findings in Cats with Confirmed Avian Influenza A/H5N1 Virus Infection During the 2023 Outbreak in Poland: A Retrospective Case Series of 22 Cats

**DOI:** 10.3390/pathogens15020200

**Published:** 2026-02-11

**Authors:** Dawid Jańczak, Anna Golke, Karol Szymański, Ewelina Hallmann, Katarzyna Pancer, Aleksander Masny, Tomasz Dzieciątkowski, Olga Szaluś-Jordanow

**Affiliations:** 1 Department of Infectious and Invasive Diseases and Veterinary Administration, Faculty of Biological and Veterinary Sciences, Institute of Veterinary Medicine, Nicolaus Copernicus University, Gagarina 7, 87-100 Toruń, Poland; 2 Department of Preclinical Sciences, Institute of Veterinary Medicine, Warsaw University of Life Sciences-SGGW, Ciszewskiego 8, 02-786 Warsaw, Poland; anna_golke@sggw.edu.pl; 3 Department of Virology, National Institute of Public Health NIH–National Research Institute, Chocimska 24, 00-791 Warsaw, Poland; kszymanski@pzh.gov.pl (K.S.); inahallmann@gmail.com (E.H.); kpancer@pzh.gov.pl (K.P.); amasny@gmx.com (A.M.); 4 Department of Medical Microbiology, Medical University of Warsaw, Chałubińskiego 5, 02-004 Warsaw, Poland; tdzieciatkowski@wum.edu.pl; 5 Department of Small Animal Diseases with Clinic, Institute of Veterinary Medicine, Warsaw University of Life Sciences-SGGW, Nowoursynowska 159c, 02-776 Warsaw, Poland

**Keywords:** avian influenza, highly pathogenic avian influenza virus, A/H5N1, cat, pneumonia, neurological signs, thrombocytopenia, serum biochemistry, Poland

## Abstract

Highly pathogenic avian influenza (HPAI) A/H5N1 has emerged as a cause of severe disease in domestic cats, but clinical data from field outbreaks remain limited. We retrospectively reviewed medical records, laboratory results, and ancillary examinations from 22 domestic cats with RT-qPCR-confirmed A/H5N1 infection diagnosed in Poland in June 2023. To the best of our knowledge, we report the first comprehensive retrospective case series from the 2023 Polish outbreak, combining 22 laboratory-confirmed cats with detailed clinical timelines and laboratory findings. For each cat, the temporal progression of clinical signs, hematology, serum biochemistry, and, when available, imaging findings were evaluated. Post-mortem examination data were not systematically available in this retrospective cohort. Notably, six of these cats were strictly indoor cats that received raw poultry meat as part of their diet. Disease onset was acute, with fever, lethargy, and anorexia rapidly progressing to severe dyspnea and neurological signs, including ataxia, seizures, and paraplegia; case fatality was 100%, with a typical interval of ≤3 days from first signs to death or euthanasia. Hematologic changes were dominated by thrombocytopenia, lymphopenia, and marked eosinopenia, consistent with a systemic inflammatory/stress leukogram. Biochemistry indicated marked tissue injury, with increased AST, LDH, and CK activities, whereas creatinine and urea remained largely within reference intervals, arguing against primary renal failure. Imaging supported the presence of interstitial to diffuse pneumonia. These data characterize the clinical and laboratory phenotypes of feline A/H5N1 infection and underscore its importance as a rapidly fatal respiratory and neurological disease with One Health implications.

## 1. Introduction

Avian influenza (also known as bird flu) is an infectious disease of birds caused by avian influenza A viruses, members of the family Orthomyxoviridae [1]. At least three hemagglutinin subtypes, H5, H7, and H9, are important pathogens in the poultry industry [2]. Wild birds serve as the natural reservoir of these viruses and have a significant impact on their global spread, including the transmission of these viruses to farmed avian species [3]. Influenza A viruses infecting poultry are divided into two pathotypes. The extremely virulent H5 and H7 subtypes cause highly pathogenic avian influenza (HPAI), with mortality that may approach 100% in susceptible flocks. In contrast, low-pathogenic avian influenza (LPAI) viruses typically cause mild respiratory disease or may even lead to subclinical infections [4]. Between 2004 and 2007 in Thailand, infection with the A/H5N1 avian influenza virus was documented in numerous wild bird species, including Anseriformes, Charadriiformes, Ciconiiformes, Columbiformes, Cuculiformes, Falconiformes, Galliformes, Gruiformes, Passeriformes, Piciformes, Psittaciformes, and Struthioniformes [3]. Infections with influenza A viruses have also been reported in several carnivorous mammals. The first descriptions involved the harbor seal (*Phoca vitulina*) [5] and the American mink (*Mustela vison*) [6]. Subsequently, fatal cases of HPAI caused by A/H5N1 virus were reported in captive tigers (*Panthera tigris*), leopards (*Panthera pardus*), domestic dogs (*Canis familiaris*), and domestic cats (*Felis catus*) [7,8,9,10].

In June–July 2023, veterinarians from various regions of Poland reported numerous cases of domestic cats presenting with severe respiratory signs, high fever, and neurological symptoms. One of the first comprehensively described cases of A/H5N1 avian influenza infection in a domestic cat in Poland was reported by Szaluś-Jordanow et al. [11], which included the clinical course, treatment, laboratory test results, and lung ultrasound. However, systematic data on the clinical and laboratory characteristics of affected cats during this outbreak remain limited. Available publications from field outbreaks have predominantly described single cases, with limited integration of hematology/biochemistry across a broader cohort. Therefore, we aimed to compile and analyze a 22-cat, laboratory-confirmed case series to better define the clinical course, laboratory abnormalities, and ancillary diagnostic findings of feline A/H5N1 infection during the 2023 outbreak in Poland.

In this study, we retrospectively analyzed 22 domestic cats with laboratory-confirmed A/H5N1 avian influenza virus infection diagnosed in Poland in 2023 (Table 1). This consolidated cohort represents a substantial expansion over previously published feline A/H5N1 clinical data, which have mainly relied on isolated case reports, and enables a more robust description of the disease timeline and laboratory signatures. For each cat, we reconstructed the clinical course from the onset of first clinical signs until death, including the evolution of respiratory and neurological manifestations and the treatments administered. Hematological and serum biochemical test results were collected and evaluated. This comprehensive dataset enables a detailed characterization of the clinical and laboratory features of A/H5N1 infection in cats.

## 2. Materials and Methods

Between 19 and 30 June 2023, veterinarians from various regions of Poland submitted biological samples (serum, EDTA-anticoagulated blood, nasal and pharyngeal swabs) to a commercial veterinary diagnostic laboratory. Samples were accompanied by medical documentation and, when available, partial results of blood tests performed earlier during the course of the disease. The medical records included information on the onset of clinical signs and the course of disease up to the time of death. However, in most cases, samples were collected and submitted on the day of the animal’s death, and HPAI infection was confirmed post-mortem.

### 2.1. Hematology

Complete blood counts were performed using various automated hematology analyzers, including Mindray BC-2800 Vet and Mindray BC-5000 Vet (Mindray Co., Ltd., Shenzhen, China).

### 2.2. Serum Biochemistry

The concentrations of selected biochemical analytes were measured by enzymatic and/or colorimetric methods using a Mindray BS-800M sequential analyzer (Mindray Bio-Medical Electronics Co., Shenzhen, China).

### 2.3. FIV/FeLV Rapid Tests

EDTA-anticoagulated blood samples were tested for the presence of feline leukemia virus (FeLV) antigens and antibodies against feline immunodeficiency virus (FIV). For this purpose, a Vet Expert Rapid Test FIV Ab/FeLV Ag (Vet Planet Sp. z o. o., Łomianki, Poland) was used.

### 2.4. Clinical Findings and Ancillary Examinations

The course of disease from the onset of the first clinical signs, together with the results of ancillary diagnostic examinations, is summarized in Table 2.

### 2.5. RNA Isolation and Real-Time PCR

To confirm A/H5N1 infection, total RNA was extracted from clinical samples using the Total RNA Mini Kit (A&A Biotechnology, Gdańsk, Poland), following the manufacturer’s instructions. Real-time reverse-transcription PCR (RT-qPCR) was then performed in duplex format with hydrolysis probes, targeting hemagglutinin and neuraminidase genes of influenza A viruses, including the A/H5N1 subtype, according to the protocol described by Stefańska et al. [12]. Primer and probe sequences, reaction mixture composition, and cycling conditions were identical to those in the original publication. Cycle threshold (Ct) values ≤ 35.00 were considered positive. Each run included a no-template control and a positive control. Sequencing and phylogenetic analysis were outside the scope of this retrospective clinical–laboratory study; therefore, no viral genomes were generated from the 22 feline cases included.

## 3. Results

### 3.1. RT-qPCR Confirmation of A/H5N1 Infection

All 22 cats met the inclusion criterion of laboratory-confirmed A/H5N1 infection by duplex RT-qPCR targeting the H5 (HA) and N1 (NA) genes (Table 3). Diagnostic material consisted predominantly of nasopharyngeal swabs (14/22), followed by pharyngeal swabs (5/22), nasal swabs (2/22), and post-mortem tissue sections (1/22). Ct values were ≤35 for both targets in all cases; the median Ct was 30.42 (IQR 29.01–33.10; range 25.40–34.42) for H5 and 31.56 (IQR 29.98–32.45; range 27.53–34.29) for N1.

### 3.2. Hematological Findings

**Figure 1:**
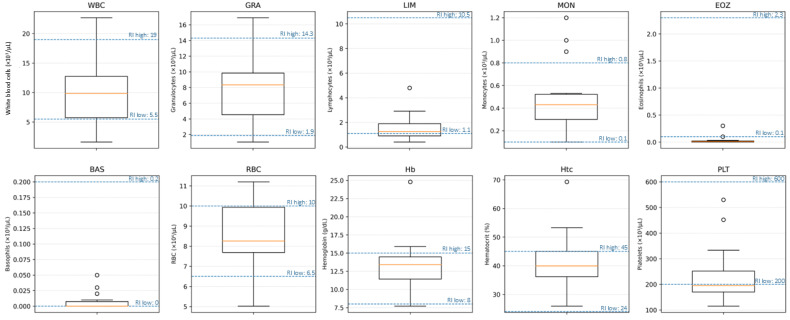
Hematological abnormalities in cats with confirmed A/H5N1 infection (*n* = 14). Boxplots show distributions of WBC, granulocytes (GRAs), lymphocytes (LIMs), monocytes (MONs), eosinophils (EOZs), basophils (BASs), RBC, hemoglobin (Hb), hematocrit (Htc), and platelets (PLTs). Dashed horizontal lines indicate the laboratory reference interval (RI) lower and upper limits for each parameter.

**Figure 2:**
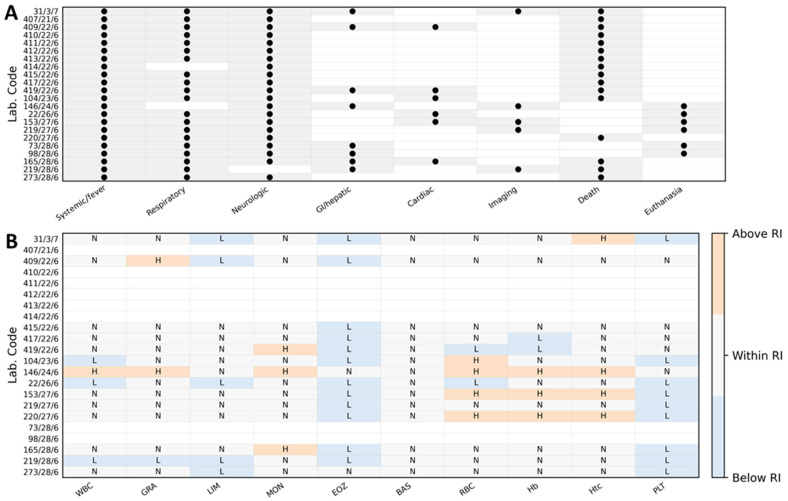
Case-level association between clinical sign categories and hematological abnormalities in cats with confirmed A/H5N1 infection. Rows correspond to individual cats identified by laboratory codes. (Panel **A**) summarizes the presence of major clinical sign categories derived from the case timelines in Table 2 (binary coding: present/absent). Categories were defined as follows: Systemic/fever—apathy/lethargy and/or anorexia with rectal body temperature above the laboratory reference threshold (when recorded); Respiratory—dyspnea, tachypnea/increased respiratory effort, abnormal lung auscultation, or imaging findings consistent with pneumonia (thoracic radiography interstitial or mixed interstitial–alveolar pattern; lung ultrasound with numerous B-lines); Neurologic—ataxia, seizures, paresis/paraplegia, stupor, cranial nerve deficits (e.g., anisocoria/miosis), torticollis, or absence of corneal reflex; Gastrointestinal/hepatic—vomiting and/or diarrhea, hypersalivation, icterus, or hepatobiliary abnormalities on ultrasound; Outcome—death or euthanasia during the acute course. (Panel **B**) shows hematological results for each cat (CBC available subset) displayed relative to the laboratory reference intervals (RI). For each parameter, values were categorized as below RI, within RI, or above RI according to the corresponding RI used by the diagnostic laboratory (RI values provided in Table 4). Abbreviations: WBC, white blood cells; GRA, granulocytes; LIM, lymphocytes; MON, monocytes; EOZ, eosinophils; BAS, basophils; RBC, red blood cells; Hb, hemoglobin; Htc, hematocrit; PLT, platelets; RI, reference interval; L, low; N, normal; H, high.

Complete blood counts were available for 14 of the 22 cats. Total leukocyte counts (WBC; RI 5.5–19.0 × 10^3^/µL) were mostly within the reference interval, with a median of 9.85 × 10^3^/µL (IQR 5.72–12.75; range 1.6–22.7 × 10^3^/µL); leukopenia was recorded in 3/14 (21%) and leukocytosis in 1/14 (7%) cats. Neutrophil counts (GRA; RI 1.9–14.3 × 10^3^/µL) showed a median of 8.35 × 10^3^/µL (IQR 4.53–9.85; range 1.05–16.9 × 10^3^/µL), with 11/14 (79%) cats within the reference interval, 1/14 (7%) neutropenic, and 2/14 (14%) neutrophilic. Lymphopenia was frequent: lymphocyte counts (LIM; RI 1.1–10.5 × 10^3^/µL) had a median of 1.25 × 10^3^/µL (IQR 0.9–1.9; range 0.4–4.8 × 10^3^/µL), and 5/14 (36%) cats were below the reference interval. Monocyte counts (MON; RI 0.1–0.8 × 10^3^/µL) were mostly normal (median 0.43 × 10^3^/µL, IQR 0.30–0.52; range 0.1–1.2 × 10^3^/µL), but 3/14 (21%) cats showed monocytosis. Eosinophil counts (EOZ; RI 0.1–2.3 × 10^3^/µL) were markedly suppressed, with a median of 0.01 × 10^3^/µL (IQR 0.00–0.02; range 0–0.3 × 10^3^/µL) and 12/14 (86%) cats below the reference interval, consistent with a stress leukogram. Basophil counts (BAS; RI 0–0.2 × 10^3^/µL) were within the reference interval in all cats (median 0.00 × 10^3^/µL, range 0–0.05 × 10^3^/µL). Red blood cell parameters were largely within or above reference intervals. RBC counts (RI 6.5–10.0 × 10^6^/µL) had a median of 8.26 × 10^6^/µL (IQR 7.69–9.94; range 5.02–11.2 × 10^6^/µL). Among the 14 cats, 2 (14%) had RBC counts below the reference interval, whereas 4 (29%) exceeded it. Hematocrit (Hct; RI 24–45%) showed a median of 40.0% (IQR 36.2–45.0; range 25.9–69.3%), with no values below the reference interval and 4/14 (29%) cats showing hemoconcentration (>45%). Hemoglobin concentration had a median of 13.4 g/dL (IQR 11.4–14.5; range 7.7–24.8 g/dL). Platelet counts (PLT; RI 200–600 × 10^3^/µL) were frequently decreased, with a median of 195 × 10^3^/µL (IQR 170–252; range 115–530 × 10^3^/µL). Thrombocytopenia (<200 × 10^3^/µL) was present in 9/14 (64%) cats, while the remaining 5/14 (36%) were within the reference interval, and no cat showed thrombocytosis. The hematological parameters evaluated in this study are summarized in Table 4. An overview of hematological abnormalities with reference intervals is provided in Figure 1. Case-level hematological abnormalities aligned with the clinical phenotype from Table 2 are summarized in Figure 2.

### 3.3. Serum Biochemical Findings—Liver-Associated Analytes

Activities of aspartate aminotransferase (AST) were markedly increased in all cats. AST exceeded the upper reference limit (44 U/L) in 22/22 (100%) animals, with a median activity of 179 U/L (IQR 138.5–339.2; range 81–8546 U/L), corresponding to a median of 4.1 times (IQR 3.1–7.7; range 1.8–194.2) the upper reference limit. Alanine aminotransferase (ALT) was more variably affected, with a median of 94.5 U/L (IQR 83.3–163.3; range 27–2445 U/L), and 10/22 (45%) cats showed activities above the reference interval (>107 U/L). Total bilirubin was increased in 12/22 (55%) cats (median 1.7 mg/dL, IQR 0.6–3.1; range 0.4–9.6 mg/dL; upper reference limit 1.2 mg/dL). In contrast, alkaline phosphatase (AP) and γ-glutamyltransferase (GGT) were less consistently elevated, with 5/22 (23%) and 8/22 (36%) cats, respectively, having values above the reference interval.

### 3.4. Serum Biochemical Findings—Muscle and Tissue Injury

Marked increases in tissue and muscle injury markers were observed in most cats. Lactate dehydrogenase (LDH; RI 70–405 U/L) was above the reference interval in 20/22 (91%) animals. LDH activity had a median value of 1248.5 U/L (IQR 669.5–1621.3; range 136–2422 U/L), corresponding to a median of approximately 3.1 times (IQR 1.7–4.0; range 0.3–6.0) the upper reference limit; 13/22 (59%) cats had LDH activities ≥3× the upper reference limit. Creatine kinase (CK; RI 70–400 U/L) was measured in 15 cats and was markedly increased in the majority of them. CK was above the reference interval in 9/15 (60%) animals, with a median of 1201 U/L (IQR 285.5–3177; range 66–7781 U/L), i.e., a median of 3.0 times (IQR 0.7–7.9; range 0.2–19.5) the upper reference limit. Very high activities compatible with severe myonecrosis (≥3× upper reference limit) were recorded in 8/15 (53%) cats.

### 3.5. Serum Biochemical Findings—Renal Parameters

**Figure 3:**
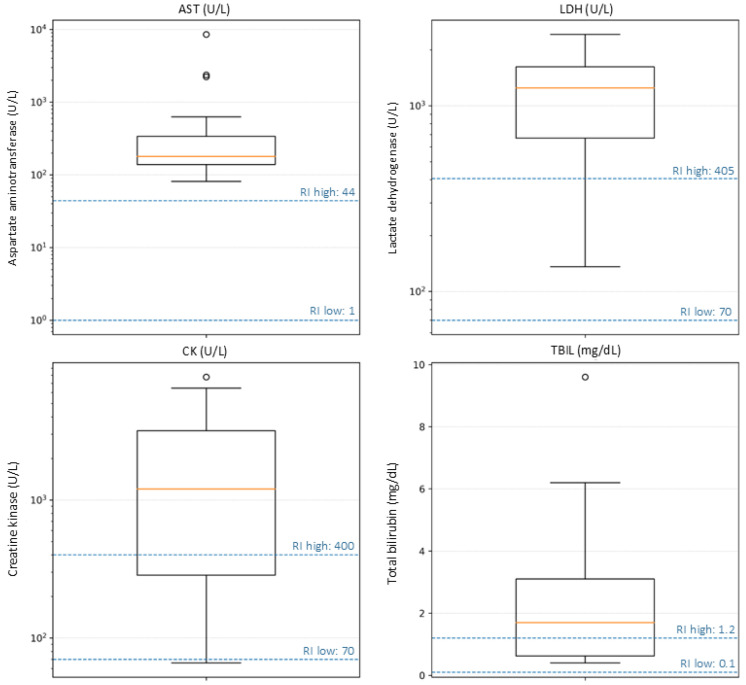
Biochemical abnormalities in cats with confirmed A/H5N1 infection. Boxplots show serum/plasma activities or concentrations of aspartate aminotransferase (AST), lactate dehydrogenase (LDH), creatine kinase (CK), and total bilirubin (TBIL). Dashed horizontal lines indicate the laboratory reference interval (RI) lower and upper limits for each analyte; AST, LDH, and CK are displayed on a logarithmic *y*-axis to accommodate the wide range of values.

In contrast, serum markers of renal function were largely within reference limits. Serum creatinine (RI 0.8–1.8 mg/dL) had a median concentration of 1.4 mg/dL (IQR 1.13–1.60; range 0.9–2.7 mg/dL); 21/22 (95%) cats had creatinine within the reference interval, and only one animal (2.7 mg/dL) showed a mild increase above the upper reference limit. Blood urea nitrogen (BUN; RI 24–70 mg/dL) had a median of 43 mg/dL (IQR 33.5–48.8; range 24–103 mg/dL), with 20/22 (91%) cats within the reference interval and only 2/22 (9%) showing mild azotemia (99 and 103 mg/dL). Overall, there was no consistent biochemical evidence of severe primary renal failure in this cohort. The serum biochemical parameters evaluated in this study are summarized in Table 5. An overview of biochemical abnormalities with reference intervals is provided in Figure 3.

### 3.6. FIV/FeLV Rapid Tests Results

FeLV antigen and anti-FIV antibodies were not detected in the blood of any of the 22 tested cats.

### 3.7. Clinical Course

**Figure 4:**
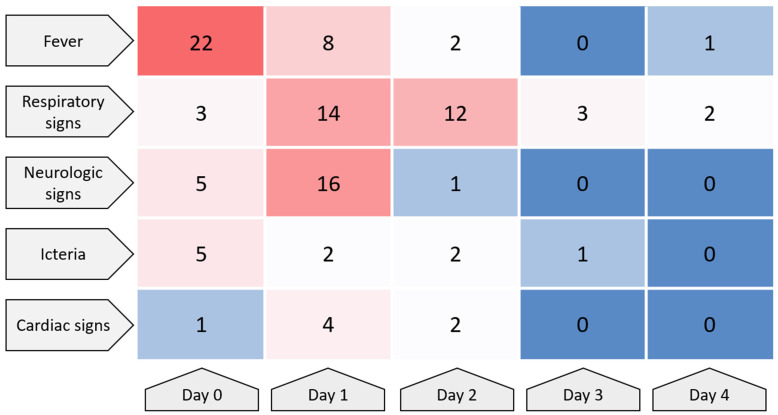
Temporal dynamics of major clinical sign categories in cats with confirmed A/H5N1 infection (*n* = 22). Heatmap showing the number of cats presenting with selected clinical sign categories from Day 0 to Day 4, where Day 0 denotes the first day of owner-observed clinical signs or the first clinical assessment recorded for each case. Cell values indicate the count of cats with the respective category documented on that day (categories are not mutually exclusive; a cat could contribute to multiple categories on the same day). Color intensity reflects frequency (higher counts shown in warmer tones and lower counts in cooler tones), illustrating the rapid onset and early predominance of respiratory and neurologic manifestations during the acute course.

**Figure 5:**
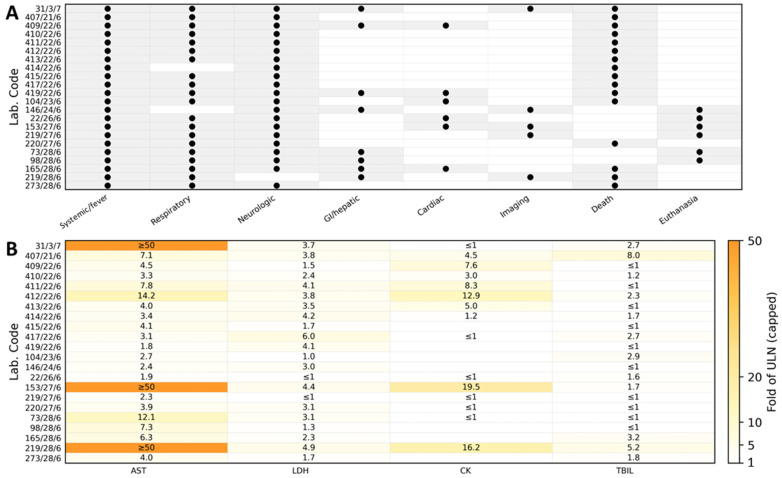
Case-level association between clinical sign categories and biochemical abnormalities expressed as fold above the upper limit of normal (ULN) in cats with confirmed A/H5N1 infection. Rows correspond to individual cats identified by laboratory codes. (Panel **A**) summarizes clinical sign categories (present/absent) derived from the timelines in Table 2 using the same definitions as in Figure 2 (Systemic/fever, Respiratory, Neurologic, Gastrointestinal/hepatic, and Outcome). (Panel **B**) displays key biochemical parameters (AST, LDH, CK, total bilirubin) aligned to the same laboratory codes and expressed as fold above ULN to facilitate comparison across cats and to accommodate wide value ranges. Fold above ULN was calculated as: fold above ULN = measured value/ULN, where ULN is the upper reference limit for the respective analyte (laboratory reference limits shown in Table 5). Values ≤ ULN were assigned a fold value ≤ 1.0. Abbreviations: AST, aspartate aminotransferase; LDH, lactate dehydrogenase; CK, creatine kinase; TBIL, total bilirubin; ULN, upper limit of normal; RI, reference interval.

According to the timelines provided by the attending veterinarians (Table 4), the disease developed rapidly in all affected cats. The first nonspecific signs, most frequently apathy, decreased appetite, and fever ≥ 39.5 °C, were usually noticed 1–2 days prior to presentation. Neurological abnormalities (ataxia, staggering gait, loss of balance, disorientation, seizures, abnormal pupillary size, and lack of menace response) and hypersalivation appeared shortly afterwards, often within 24 h of the first clinical signs. In parallel, 20 out of 22 cats developed progressive respiratory signs, including tachypnoea, dyspnea, and increased abdominal effort; in several animals, pulmonary interstitial or alveolar changes were confirmed by thoracic imaging or lung ultrasound. Based on the available timelines, the overall clinical course was fulminant: in most cats, the interval from the first observed signs to death or euthanasia did not exceed 2–3 days. In the majority of animals, death or euthanasia occurred on the day of, or the day after, the first veterinary examination, despite supportive treatment. Figure 4 illustrates the temporal distribution of cases, showing the number of cats presenting with selected clinical sign categories from Day 0 to Day 4. Case-level association between clinical sign categories and biochemical abnormalities are summarized in Figure 5.

## 4. Discussion

During the 2022/23 epidemic season in Poland, highly pathogenic avian influenza (HPAI) A(H5N1) was widely detected in both poultry and wild birds, reflecting sustained viral circulation and repeated introductions from avian reservoirs [13]. Importantly, in late June–early July 2023—concurrent with the cluster of feline cases—additional HPAI events in poultry were reported, and official investigations commonly implicated indirect contact with wild birds as the most likely route of introduction into backyard holdings [14]. Published genomic analyses of viruses detected in cats during the same outbreak further demonstrated clustering within clade 2.3.4.4b and close relatedness to contemporaneously detected avian H5N1 viruses in the region, supporting an avian origin of infection and a common exposure context [13,15].

This retrospective study describes a cluster of 22 domestic cats with confirmed A/H5N1 infection in Poland in June 2023, with particular emphasis on respiratory involvement. Importantly, this case series extends the existing evidence base beyond the predominantly single-case clinical reports published to date and provides an integrated overview of clinical progression, including hematology and serum biochemistry, in a larger, laboratory-confirmed cohort. The clinical picture was dominated by an acute onset of fever, depression, and anorexia, rapidly followed by severe respiratory distress and neurological abnormalities. Imaging, supported the presence of pneumonia. The overall clinical course was fulminant, with most cats dying or being euthanized within a few days of the onset of clinical signs. These observations reaffirm the strong respiratory tropism and high virulence of contemporary HPAI A/H5N1 viruses in felids, complementing previous reports from Asia, Europe, and North America [16,17,18,19]. In experimental and natural infections, A/H5N1 has consistently caused severe lower respiratory tract disease in cats, characterized clinically by dyspnea, tachypnea, and rapid progression to respiratory failure, and histologically by diffuse or bronchointerstitial pneumonia with extensive alveolar damage [16,17,20]. In our cohort, dyspnea and increased respiratory effort were among the most frequently recorded clinical signs by veterinarians, and thoracic imaging documented interstitial or mixed interstitial–alveolar lung patterns, as well as partial loss of aeration. Lung ultrasound repeatedly showed numerous B-lines, consistent with interstitial–alveolar syndrome. These findings are consistent with published descriptions of A/H5N1-associated pulmonary lesions in cats and other carnivores, in which interstitial pneumonia and diffuse alveolar damage constitute the predominant pathological changes [17,18,19]. In contrast to A/H5N1, most other influenza A infections in cats tend to be milder and predominantly affect the upper respiratory tract. Reviews and guidelines indicate that feline influenza is generally rare worldwide and typically presents as an acute but self-limiting upper respiratory disease, characterized by sneezing, nasal discharge, and conjunctivitis as the primary signs; pneumonia is often absent or attributed to secondary bacterial infection [21]. Outbreaks caused by other subtypes, such as pandemic A/H1N1 or low-pathogenic avian A/H7N2, in shelter cats have been associated with variable severity of respiratory disease, including cases of pneumonia, but with markedly lower case fatality rates than those observed with A/H5N1 [22,23]. By contrast, A/H5N1 infections in felids are frequently fatal and are characterized by systemic dissemination with primary involvement of the lungs and central nervous system [16,24]. The 100% fatality rate (22/22 in our series) and the peracute progression from non-specific signs to respiratory failure mirror these earlier observations and underscore that A/H5N1-associated pneumonia in cats should be regarded as a life-threatening condition rather than a benign respiratory infection. The pattern of respiratory involvement observed in our cats, characterized by rapidly progressive dyspnea and imaging evidence of interstitial–alveolar lung disease, is also consistent with current histopathological data from recent A/H5N1 outbreaks in North America and Europe. In naturally infected cats, interstitial pneumonia, alveolar oedema, fibrinous exudation, and hyaline membranes are frequently reported, often accompanied by viral antigen in type II pneumocytes and macrophages [17,18,19]. These lesions are compatible with an acute respiratory distress syndrome (ARDS)-like process. However, the lack of systematic necropsy and histopathology in this retrospective cohort limits our ability to confirm the underlying pathomechanisms responsible for the observed respiratory failure, such as diffuse alveolar damage or bronchointerstitial pneumonia. Therefore, any inferences regarding pulmonary pathology are based primarily on the clinical phenotype and the available imaging findings, rather than on tissue-level confirmation. From a pathophysiological perspective, the preferential replication of A/H5N1 in the lower respiratory tract, combined with intense local cytokine responses and vascular injury, likely contributes to the abrupt onset of life-threatening respiratory compromise in affected cats, as has been described in experimental studies [20].

The hematological and biochemical findings observed in our cohort further support the concept of severe systemic inflammation, characterized by prominent pulmonary and extrapulmonary tissue damage. Thrombocytopenia was the most consistent hematological abnormality, and lymphopenia accompanied by marked eosinopenia was also common, consistent with a stress or systemic inflammatory leukogram. Similar patterns have been described in experimental A/H5N1 infection in cats and in naturally infected carnivores, where thrombocytopenia has been interpreted as a marker of consumptive coagulopathy and endothelial damage [17,19,20]. In serum biochemistry, activities of AST, LDH, and CK were markedly elevated in most animals, whereas creatinine and urea were comparatively less affected. This pattern suggests predominant injury of the lung, liver, and skeletal or respiratory musculature with limited primary renal involvement. High AST and LDH concentrations have also been documented in the blood or ocular fluids of A/H5N1-infected cats in earlier European reports [10,16,19]. Together with the clinical and imaging features, these findings support the interpretation that A/H5N1 infection in our cats caused a predominantly respiratory and systemic inflammatory disease, rather than a multi-organ failure dominated by renal dysfunction.

Although none of the individual hematological or biochemical abnormalities observed in this cohort are pathognomonic for H5N1 infection, their value lies in the combined clinical–laboratory pattern and its peracute evolution in a molecularly confirmed outbreak setting. In particular, the rapid progression from fever and non-specific systemic signs to severe respiratory compromise—often accompanied by neurological manifestations—together with a profile of marked tissue injury markers (elevated AST, LDH and/or CK) and frequent hyperbilirubinemia may help clinicians recognize a compatible syndrome. In an appropriate epidemiological situation like outdoor access, exposure to birds, or raw poultry feeding, this pattern should trigger prompt RT-qPCR testing for A/H5N1 and immediate infection-control measures, rather than being interpreted as a non-specific viral infection. This perspective is particularly relevant for practitioners managing cats with severe pneumonia of suspected viral origin.

When interpreted within the broader context of viral pneumonia in cats, the present data highlight several important points for veterinary practitioners. Primary viral pneumonia in cats is relatively uncommon or may be underdiagnosed. Most feline lower respiratory tract diseases are associated with bacterial infection, parasitic disease, aspiration, or neoplasia, whereas classic viral pathogens of the feline upper respiratory tract (Feline Herpesvirus-1, Feline Calicivirus) typically cause rhinitis, conjunctivitis, and tracheobronchitis, with pneumonia mainly arising as a complication or in shelters and multi-cat environments [25].

Influenza A viruses thus represent a relatively rarely diagnosed but clinically significant cause of acute pneumonia, occurring exclusively when highly pathogenic strains are involved. The combination of acute onset, very high fever, marked dyspnea, and concurrent neurological signs, as observed here, is atypical for the usual feline respiratory disease complex and should promptly raise suspicion of HPAI infection, particularly in cats with exposure to wild birds or poultry or their feces or raw meat. In our study, raw poultry feeding was documented in 13/22 (59%) affected cats, and 6 of them were strictly indoor cats, indicating that exposure to raw poultry meat may have been an important route of infection in this cluster. Nevertheless, it was not possible to test poultry meat for the presence of A/H5N1 virus; therefore, it is only an observation.

Our data also provides some practical insights into diagnostic approaches for feline patients presenting with severe pneumonia of suspected viral origin. In most cases, samples for RT-qPCR were collected late in the course of disease, often on the day of death. Earlier sampling of nasal or pharyngeal swabs, and, where feasible, tracheal or lung samples, could facilitate ante-mortem confirmation and timely implementation of infection-control measures. Current guidelines recommend molecular diagnosis of influenza in cats with acute respiratory signs and relevant exposure history, although routine testing is not indicated in uncomplicated upper respiratory infections [21]. Lung ultrasound, which in our series frequently revealed numerous B-lines, may prove to be a useful tool to detect interstitial–alveolar syndrome and monitor progression in severely dyspneic cats, but these sonographic findings are non-specific and cannot distinguish influenza from other causes of viral or non-viral pneumonia.

From a One Health perspective, the present cluster also contributes to the growing body of evidence that domestic cats are susceptible spillover hosts for contemporary clade 2.3.4.4b A/H5N1 viruses circulating in wild birds and poultry [19,26,27,28]. Previous outbreaks of A/H5N1 in tigers, leopards, and domestic cats, as well as the A/H7N2 shelter outbreak in New York with documented cat-to-human transmission, illustrate that felids can both suffer severe disease and, in some circumstances, participate in onward transmission [16,23,24]. Although zoonotic transmission from cats appears to be rare, the high viral loads in severely affected animals and the close contact between cats and humans justify strict adherence to biosafety recommendations when managing suspected or confirmed cases, including isolation, use of personal protective equipment, and appropriate handling of carcasses, as outlined in existing guidelines [19,21].

This study has several limitations that should be considered when interpreting the findings. The retrospective design and reliance on referring veterinarians’ documentation resulted in incomplete datasets for some cats, particularly regarding imaging, histopathology, and potential co-infections. An important limitation is that no poultry, raw-meat, or environmental samples linked to the affected households were available for testing. Therefore, the putative exposure source and transmission route cannot be confirmed for this case series [14]. Consequently, our conclusions regarding exposure pathways rely on owner history (including reported raw poultry feeding), the temporal overlap with HPAI activity in Poland, and the published molecular context from the broader 2023 feline outbreak rather than direct source attribution [13,14,15]. The number of animals is modest, and the study population may not represent the full clinical spectrum of feline A/H5N1 infection, as mild or subclinical cases would be unlikely to be sampled [29]. No systematic bacteriological investigations of the lower respiratory tract were performed, so the contribution of secondary bacterial pneumonia cannot be fully ruled out. Nevertheless, the peracute course, the extent of respiratory and neurological signs, and the high fatality rate strongly suggest that A/H5N1 itself was the principal infectious agent of disease in this outbreak.

Although molecular characterization of the virus was not performed in the present study, we acknowledge the lack of sequencing data as a limitation of this retrospective case series; whenever sufficient archived material is available, future prospective studies will include the sequencing of representative samples to support the molecular characterization of feline H5N1 viruses. Publicly available sequence data from the same feline outbreak in Poland provide important contextual information. Rabalski et al. reported the deposition of feline A/H5N1 sequences in GISAID, including EPI_ISL_17949824 (A/cat/Poland/Gda1/2023; whole-genome sequence) and EPI_ISL_17989196 (A/cat/Poland/Kra1/2023; partial genome with complete HA and NA segments) [15]. Furthermore, Domańska-Blicharz et al. demonstrated that viruses detected in cats during the 2023 Polish outbreak clustered within clade 2.3.4.4b and were closely related to avian H5N1 viruses circulating in wild birds in the region [13]. Together, these findings support a common avian source of exposure for feline infections, although detailed molecular analyses were beyond the scope of the present clinical study.

## 5. Conclusions

Our findings confirm that A/H5N1 infection in domestic cats is associated with highly fatal, rapidly progressive respiratory and neurological disease, in which pneumonia and acute respiratory failure are central features. The clinical pattern, observed acute onset, fever, and depression, followed within 1–3 days by severe dyspnea, radiographic or sonographic evidence of lung involvement, and frequent neurological signs, should alert veterinarians to the possibility of HPAI infection, especially in cats with outdoor access, exposure to raw poultry meat, or contact with wild birds or their feces. Early recognition, targeted diagnostics, and the implementation of appropriate biosafety measures are essential for potential zoonotic and epidemiological risks.

## Figures and Tables

**Table 1:** Signalment, lifestyle characteristics, and diet of cats with confirmed A/H5N1 infection.

Lab. Code	Voivodeship	Age	Sex	Outdoor Access	Raw Meat in the Diet
31/3/7	Pomeranian	11 Y 1 M	M	no	poultry, chicken
407/21/6	Kuyavian-Pomeranian	6 Y	F	yes	poultry, chicken
409/22/6	Lower Silesia	5 Y	M	yes	poultry, chicken, pork
410/22/6	Pomeranian	n/a	M	yes	n/a
411/22/6	Pomeranian	n/a	M	yes	n/a
412/22/6	Pomeranian	n/a	M	yes	n/a
413/22/6	Pomeranian	n/a	F	yes	n/a
414/22/6	Pomeranian	n/a	M	yes	n/a
415/22/6	Podlaskie	2 Y	F	yes	n/a
417/22/6	Mazovian	2 M	M	no	poultry, chicken
419/22/6	Mazovian	1 Y 6 M	M	no	poultry, chicken breast
104/23/6	Kuyavian-Pomeranian	2 Y 6 M	F	yes	poultry, chicken liver
146/24/6	Kuyavian-Pomeranian	3 Y	M	yes	n/a
22/26/6	Opole	4 Y	F	no	pork
153/27/6	Greater Poland	2 Y	M	no	poultry, chicken
219/27/6	Greater Poland	5 Y 6 M	F	no	poultry, chicken liver
220/27/6	Kuyavian-Pomeranian	3 Y	M	no	poultry, chicken, turkey
73/28/6	Silesian	4 Y 6 M	M	yes	poultry, chicken breast
98/28/6	Lower Silesia	12 Y	M	yes	poultry, chicken
165/28/6	Warmian-Masurian	6 Y 3 M	M	yes	poultry, chicken liver
219/28/6	Łódź	3 Y	M	yes	poultry, chicken, beef
273/28/6	Mazovian	10 Y	M	yes	n/a

**Table 2:** The course of disease from the onset of the first clinical signs to death/euthanasia in 22 cats.

Lab. Code	16 June 2023	17 June 2023	18 June 2023	19 June 2023	20 June 2023	21 June 2023	22 June 2023	23 June 2023	24 June 2023
31/3/7	-	-	Clinical signs observed by the owner: loss of appetite and lethargy.	→		Clinical findings: apathy, dehydration, BT 40.6 °C	dementia, ataxia	thoracic X-ray: diffuse interstitial pattern, peribronchial opacities; abdominal US: dilated bile ducts, thickened bile duct/gallbladder wall; dyspnea; death	-
407/21/6	-	Clinical findings: apathy, lethargy, BT 40 °C	→	miosis, anisocoria, ataxia	dyspnea, death	-	-	-	-
409/22/6	Clinical signs observed by the owner: apathy, loss of appetite, vomiting	→				Clinical findings: BT 40.6 °C, initially miosis, binocular anisocoria, right torticollis, tachycardia (HR 150/min), dyspnea, death	-	-	-
410/22/6	Clinical findings: BT 41 °C, apathy, lethargy, dyspnea	binocular anisocoria, ataxia, paraplegia	severe dyspnea, death	-	-	-	-	-	-
411/22/6	Clinical findings: apathy, loos of appetite, BT 40 °C, anisocoria	severe dyspnea, death	-	-	-	-	-	-	-
412/22/6	-	Clinical findings: salivation, anisocoria, ataxia, BT 40.2 °C	limb muscle stiffness, dyspnea, death	-	-	-	-	-	-
413/22/6	Clinical signs observed by the owner: loss of appetite	→	Clinical findings: BT 39.7 °C, dyspnea, binocular anisocoria	severe dyspnea, death	-	-	-	-	-
414/22/6	-	Clinical signs observed by the owner: lethargy, apathy, salivation	→	Clinical findings: BT 41 °C, seizures, paraplegia, death	-	-	-	-	-
415/22/6	-	-	-	-	-	-	Clinical findings: apathy, loss of appetite, BT 40.5 °C	paresis, ataxia, paraplegia, salivation, dyspnea, miosis	severe dyspnea, death
417/22/6	-	Clinical signs observed by the owner: apathy, loss of appetite, salivation	→				Clinical findings: BT 41 °C, lethargy, binocular anisocoria, ataxia, dyspnea	severe dyspnea, death	
419/22/6	-	-	-	-	-	Clinical findings: BT 40.5 °C, apathy, ataxia, binocular anisocoria	dyspnea, tachycardia (HR 210/min), salivation	→	severe dyspnea, death
104/23/6		Clinical signs observed by the owner: apathy, loss of appetite, lethargy	→		Clinical findings: seizures, binocular anisocoria, BT 41 °C, ataxia, paraplegia	dyspnea, tachycardia, death			
146/24/6	-	-	-	-	Clinical findings: BT 38.6 °C, vomiting, diarrhea, salivation	miosis, anisocoria, ataxia	homogeneous liver parenchyma with rounded margins and altered contour; hyperechoic hepatic capsule; euthanasia due to poor prognosis	-	-
22/26/6	-	-	-	-	-	Clinical findings: BT 39.8 °C, apathy, lethargy, loss of appetite, paraplegia, binocular anisocoria, lack of corneal reflex	dyspnea, tachycardia (HR 190/min)	euthanasia due to poor prognosis	-
153/27/6	Clinical findings: BT 39.6, loss of appetite, apathy, tracheal and alveolar murmur, lethargy	→					ataxia, paraplegia, binocular anisocoria, dyspnoea, tachycardia, lung US: numerous B-lines	severe dyspnea, euthanasia due to poor prognosis	-
219/27/6	Clinical findings: BT 39.1, apathy, lethargy, loss of appetite	→				seizures, lack of corneal reflex, ataxia, limb muscle stiffness, dyspnea, stupor	severe dyspnea, lung US: numerous B-lines, euthanasia due to poor prognosis	-	-
220/27/6	-	-	-	-	-	Clinical signs observed by the owner: apathy, lethargy	Clinical findings: BT 39.8 °C, limb muscle stiffness, dyspnea, binocular anisocoria, seizures, paraplegia	severe dyspnea, death	-
73/28/6	-	-	-	-	-	-	-	Clinical findings: lethargy, vomiting, BT 38.9 °C	BT 39.7 °C, seizures, dyspnea, miosis, 3 days later euthanasia due to poor prognosis
19.98.28.6	-	-	-	-	-	-	-	-	Clinical findings: BT 37.4 °C, seizures, paraplegia, ataxia, binocular anisocoria, limb muscle stiffness, severe dyspnea (RR 38/min), tachycardia (HR 154/min), failed resuscitation, death
20.165.28.6	-	-	-	-	-	Clinical findings: loos of appetite, lethargy, salivation, BT 39.6 °C	seizures, ataxia, binocular anisocoria, right torticollis, photophobia	tachycardia (HR 152/min), dyspnea (RR 36/min)	severe dyspnea, failed resuscitation, death
21.219.28.6	-	-	Clinical findings: BT 40.5 °C, loos of appetite, painful abdominal cavity	→	vomiting, salivation, tracheal murmur	lethargy, BT 41 °C, icterus	thoracic X-ray: pulmonary oedema; lungs only partially aerated	severe dyspnea, death	
22.273.28.6	-	-	-	-	-	-	-	Clinical findings: BT 38.6, apathy, lethargy, loss of appetite	stupor, seizures, lack of corneal reflex, binocular anisocoria, dyspnea, death on the next day

The arrow (→) indicates chronological progression of clinical status between consecutive time points/visits, from earlier to later observations; a dash (-) indicates that data were not recorded for a given time point.

**Table 3:** Duplex RT-qPCR confirmation of A/H5N1 infection in 22 cats.

Lab. Code	Specimen for RT-qPCR	H5 Ct	N1 Ct	Interpretation *
31/3/7	nasopharyngeal swab	34.42	32.97	Positive (H5+/N1+)
407/21/6	nasopharyngeal swab	29.87	31.65	Positive (H5+/N1+)
409/22/6	pharyngeal swab	30.43	31.78	Positive (H5+/N1+)
410/22/6	nasopharyngeal swab	33.67	32.20	Positive (H5+/N1+)
411/22/6	nasopharyngeal swab	30.40	31.58	Positive (H5+/N1+)
412/22/6	nasopharyngeal swab	33.21	31.53	Positive (H5+/N1+)
413/22/6	nasopharyngeal swab	29.11	30.52	Positive (H5+/N1+)
414/22/6	nasopharyngeal swab	28.22	30.55	Positive (H5+/N1+)
415/22/6	nasopharyngeal swab	31.08	32.80	Positive (H5+/N1+)
417/22/6	nasopharyngeal swab	28.98	28.78	Positive (H5+/N1+)
419/22/6	nasopharyngeal swab	33.30	32.99	Positive (H5+/N1+)
104/23/6	nasopharyngeal swab	27.17	29.80	Positive (H5+/N1+)
146/24/6	tissue sections (lungs)	27.91	28.33	Positive (H5+/N1+)
22/26/6	nasal swab	30.40	32.29	Positive (H5+/N1+)
153/27/6	pharyngeal swab	30.72	31.30	Positive (H5+/N1+)
219/27/6	nasal swab	29.97	29.53	Positive (H5+/N1+)
220/27/6	pharyngeal swab	33.41	34.29	Positive (H5+/N1+)
73/28/6	nasopharyngeal swab	34.11	33.10	Positive (H5+/N1+)
98/28/6	pharyngeal swab	25.40	27.89	Positive (H5+/N1+)
165/28/6	nasopharyngeal swab	32.78	32.50	Positive (H5+/N1+)
219/28/6	pharyngeal swab	25.92	27.53	Positive (H5+/N1+)
273/28/6	nasopharyngeal swab	31.75	30.96	Positive (H5+/N1+)

* Interpretation: Ct ≤ 35.00 for the H5 (HA) and/or N1 (NA) target was considered positive, according to the protocol of Stefańska et al. All cases in this cohort were positive for both targets (H5+/N1+).

**Table 4:** Tested hematological parameters and reference intervals (RIs).

	WBC	GRA	LIM	MON	EOZ	BAS	RBC	Hb	Htc	PLT
Lab. Code	5.5–19 × 10^3^/µL	1.9–14.3 × 10^3^/µL	1.1–10.5 × 10^3^/µL	0.1–0.8 × 10^3^/µL	0.1–2.3 × 10^3^/µL	0–0.2 × 10^3^/µL	6.5–10 × 10^6^/µL	8–15 g/dL	24–45%	200–600 × 10^3^/µL
31/3/7	5.7	4.61	0.82	0.23	0	0	9.06	14.5	46.3	168
407/21/6	n/t	n/t	n/t	n/t	n/t	n/t	n/t	n/t	n/t	n/t
409/22/6	16.3	14.9	0.9	0.5	0.01	0	8.27	13.5	35.42	452
410/22/6	n/t	n/t	n/t	n/t	n/t	n/t	n/t	n/t	n/t	n/t
411/22/6	n/t	n/t	n/t	n/t	n/t	n/t	n/t	n/t	n/t	n/t
412/22/6	n/t	n/t	n/t	n/t	n/t	n/t	n/t	n/t	n/t	n/t
413/22/6	n/t	n/t	n/t	n/t	n/t	n/t	n/t	n/t	n/t	n/t
414/22/6	n/t	n/t	n/t	n/t	n/t	n/t	n/t	n/t	n/t	n/t
415/22/6	9.9	8.3	1.1	0.5	0	0.01	7.45	11.2	38.5	333
417/22/6	5.8	4.5	1.2	0.1	0.01	0	8.16	7.7	39	261
419/22/6	11.3	8.7	1.3	1.2	0.02	0.03	5.02	7.8	25.9	225
104/23/6	5.4	2.07	2.91	0.36	0.02	0.05	10.23	14.2	43.4	115
146/24/6	22.7	16.9	4.8	0.9	0.1	0	10.5	15.1	45.5	530
22/26/6	5.3	4.1	0.9	0.3	0	0	6.3	14.4	39.5	128
153/27/6	13.3	11.6	1.4	0.3	0	0	10.4	15.9	53.3	177
219/27/6	9.8	7.32	1.88	0.53	0.03	0.02	7.62	12.1	29.9	196
220/27/6	12.6	9.7	2.6	0.3	0	0	11.2	24.8	69.3	190
73/28/6	n/t	n/t	n/t	n/t	n/t	n/t	n/t	n/t	n/t	n/t
98/28/6	n/t	n/t	n/t	n/t	n/t	n/t	n/t	n/t	n/t	n/t
165/28/6	12.8	9.9	1.9	1	0.02	0	8.75	12.1	40.4	194
219/28/6	1.6	1.05	0.40	0.1	0.01	0	8.25	13.3	43.5	196
273/28/6	9.8	8.4	0.6	0.5	0.3	0	7.9	10	33	124
Median	9.8	8.35	1.25	1.25	0.01	0	8.26	13.4	40	195
IQR	5.7–12.8	4.53–9.85	0.9–1.9	0.9–1.9	0–0.02	0–0.01	7.69–9.94	11.2–14.5	37–44.5	170–252
min–max	1.6–22.7	1.05–16.9	0.4–4.8	0.4–4.8	0–0.3	0–0.05	5.02–11.2	7.7–24.8	25.9–69.3	115–530
<RI	3/14	1/14	5/14	5/14	12/14	0/14	2/14	2/14	0/14	9/14
=RI	10/14	11/14	9/14	9/14	2/14	14/14	8/14	9/14	10/14	5/14
>RI	1/14	2/14	0/14	0/14	0/14	0/14	4/14	3/14	4/14	0/14

n/t—not tested.

**Table 5:** Tested biochemical parameters and reference intervals (RIs).

	GLU	ALT	AST	AST/ALT	AP	LDH	GGTP	T. BILIR.	CK	KREA	UREA	T. PROT.	ALB	GLO	A/G
Lab. Code	100–130 mg/dL	1–107 U/L	1–44 U/L	0–1.1	11–49 U/L	70–405 U/L	0–10 U/L	0.1–1.2 mg/dL	70–400 U/L	0.8–1.8 mg/dL	24–70 mg/dL	6–7.9 g/dL	2.5–3.9 g/dL	2.6–5.1 g/dL	≥0.6
31/3/7	147	2445	8546	3.5	21	1499	2.0	3.2	235	1.5	32	8.6	3.1	5.5	0.6
407/21/6	132	477	311	0.7	80	1539	9.0	9.6	1803	2.7	103	7.0	3.2	3.8	0.8
409/22/6	212	112	199	1.8	24	594	1.0	0.4	3052	1.8	47.8	7.8	3.3	4.5	0.7
410/22/6	123	84	146	1.7	34	963	7.0	1.5	1201	1	99	7.3	2.9	4.4	0.7
411/22/6	100	83	345	4.2	17	1656	4.0	0.6	3302	1.4	49	7.3	3.0	4.3	0.7
412/22/6	145	178	627	3.5	47	1550	19	2.8	5180	1.1	64	7.6	2.8	4.8	0.6
413/22/6	122	88	175	2.0	16	1405	2.0	1.0	2018	1.8	38	7.1	3.0	4.1	0.7
414/22/6	131	164	150	0.9	57	1682	18	2	491	1.1	43	7.4	3.2	4.2	0.8
415/22/6	133	70	181	2.6	31	671	1.0	0.6	n/t	1.	42	8.6	3.5	5.1	0.7
417/22/6	189	131	136	1.0	24	2422	12	3.2	286	1.5	54	9.7	3.1	6.6	0.5
419/22/6	162	92	81	0.9	36	1645	0.0	0.5	n/t	1.7	47	7.5	3.4	4.1	0.8
104/23/6	218	51	119	2.3	26	421	2.0	3.5	n/t	1.6	25	7.4	2.9	4.5	0.6
146/24/6	137	88	106	1.2	32	1232	3.0	0.8	n/t	1.2	43	8.5	3.8	4.7	0.8
22/26/6	190	27	82	3.0	26	136	4.0	1.9	285	1.6	43	7.3	2.8	4.5	0.6
153/27/6	154	371	2229	6.0	21	1770	16	2	7781	1.8	48	6.7	2.2	4.5	0.5
219/27/6	360	70	101	1.4	11	190	1.0	0.4	336	1.4	70	8.0	3.4	4.6	0.7
220/27/6	367	96	171	1.8	16	1253	17	0.9	66	1.1	24	7.9	3.9	4.0	1.0
73/28/6	177	347	533	1.5	11	1244	12	0.4	216	1.3	28	8	2.7	5.3	0.5
98/28/6	135	144	322	2.2	67	516	15	0.7	n/t	1.4	33	8.8	3.8	5	0.8
165/28/6	179	161	276	1.7	17	936	13	3.9	n/t	1.3	35	7.3	2.9	4.4	0.7
219/28/6	190	49	2374	48.4	152	2003	2.0	6.2	6467	1.2	25	7.6	3.3	4.3	0.8
273/28/6	166	93	177	1.9	50	669	7.0	2.1	n/t	0.9	37	7.6	2.5	5.1	0.5
Median	158	94.5	179	1.85	26	1248.5	5.5	1.7	1201	1.4	43	7.6	3.1	4.5	0.7
IQR	133–190	83–163	138.5–339.3	1.4–3.0	18–44	669–1645	2–13	0.63–3.10	285.5–3177	1.1–1.6	34–48.5	7.3–8.0	2.9–3.4	4.3–5.0	0.6–0.8
min–max	100–367	27–2445	81–8546	0.7–48.4	11–152	136–2422	0–19	0.4–9.6	66–7781	0.9–2.7	24–103	6.7–9.7	2.2–3.9	3.8–6.6	0.5–1
<RI	0	0	0	0	0	0	0	0	1	0	0	0	1	0	4/22
=RI	3/22	12/22	0	4	17/22	2/22	14/22	10/22	5/15	21/22	20/22	15/22	21/22	19/22	18/22
>RI	19/22	10/22	22/22	18/22	5/22	20/22	8/22	12/22	9/15	1/22	2/22	7/22	0/22	3/22	0/22

n/t—not tested.

## Data Availability

The original contributions presented in this study are included in this article. Further inquiries can be directed to the corresponding authors. No novel sequence data were generated in this study. Representative A/H5N1 sequences from the 2023 Polish feline outbreak are publicly available in the GISAID database (e.g., EPI_ISL_17949824 and EPI_ISL_17989196).
